# Supplementary material for: Comparative analysis of temperature effect on bandgap characteristics in 1D phononic crystals: Periodic versus quasiperiodic structures
Source: PLoS One. 2026 Jun 1;21(6):e0349944. doi: 10.1371/journal.pone.0349944 (PMC13225373; doi:10.1371/journal.pone.0349944)
Supplement: S1 Table — (DOCX) [file pone.0349944.s001.docx]

S1 Table. The statistical distribution of sensitivity magnitudes derived from 14 temperature data points, with sensitivity calculated for each configuration.

| Sensitivity Range (Hz/K) | Perfect | Fibonacci | Thue-Morse | Double-Periodic | Cantor |
| --- | --- | --- | --- | --- | --- |
| -1200 to -1100 | 0 | 1 | 0 | 0 | 0 |
| -1100 to -1000 | 0 | 0 | 0 | 0 | 0 |
| -1000 to -900 | 0 | 9 | 0 | 0 | 0 |
| -900 to -800 | 0 | 0 | 0 | 0 | 4 |
| -800 to -700 | 3 | 3 | 3 | 0 | 0 |
| -700 to -600 | 0 | 0 | 0 | 4 | 9 |
| -600 to -500 | 13 | 1 | 0 | 0 | 0 |
| -500 to -400 | 0 | 0 | 11 | 9 | 1 |
| -400 to -300 | 0 | 0 | 0 | 0 | 0 |
| -300 to -200 | 0 | 0 | 0 | 1 | 0 |
| -200 to -100 | 0 | 0 | 0 | 0 | 0 |
| -100 to 0 | 0 | 0 | 0 | 0 | 0 |
| 0 to 100 | 1 | 0 | 0 | 0 | 0 |
