# Supplementary material for: Comparative analysis of temperature effect on bandgap characteristics in 1D phononic crystals: Periodic versus quasiperiodic structures
Source: PLoS One. 2026 Jun 1;21(6):e0349944. doi: 10.1371/journal.pone.0349944 (PMC13225373; doi:10.1371/journal.pone.0349944)
Supplement: Attachment — Submitted filename: Response to Reviewers.docx [file pone.0349944.s003.docx]

**Authors' Response to Reviewers’ comments**

**Manuscript Number: PONE-D-26-00953**

**Title: Comparative Analysis of Temperature Effect on Bandgap Characteristics in 1D Phononic Crystals: Periodic Versus Quasiperiodic structures**

**Journal: PLOS One**

To

The Editor,

Respected Professor,

The authors would like to sincerely thank the reviewers for their meticulous reading of the manuscript and making valuable suggestions for enriching and improving the quality and importance of the manuscript for the scientific community. Authors do believe that the revisions made in the final version of the manuscript have a significant contribution from such sincere and constructive review of the reviewer. Therefore, in submission of gratitude and respect, authors acknowledge the anonymous reviewer. The comments and suggestions are highly appreciated and included in the modified version of the manuscript. All the changes made by the authors are highlighted in red colour within the revised manuscript. Also, itemized responses to the specific questions/queries raised by the reviewers are presented below, for clarification.

With best regards:

Prof: Hussein A. Elsayed

The corresponding Author

**Response to Reviewer 1's Comments:**

This manuscript presents a Terfenol-D–based phononic crystal for magnetic-field sensing. The concept is novel, and I believe only minor revisions are needed for publication in Materials Technology: Advanced Performance Materials.

**1. Please review the text to ensure that acronyms are not defined twice (e.g., PnC). Also, once an acronym is defined, it should be used (e.g., PnBG)?**

We are thankful to the reviewer for this suggestion.

We want to clarify that the two Terfenol-D layers, which sandwich PMMA layer, are different in terms of their thickness.

The prime reason for selecting different thicknesses is that the Terfenol-D has high density, high elastic modulus, and magnetostrictive behavior, making its layer thickness an important design parameter. Due to the two Terfenol-D materials of different thickness, the acoustic path length per period is modified, which leads to change the Bragg scattering condition (λ = 2Λ, where Λ is the unit-cell size). This shifts the bandgap frequency. A thicker Terfenol-D layer increases the effective acoustic mass, lowering the bandgap frequency, while a thinner layer results in a higher bandgap. Additionally, thickness-dependent stress and magnetostrictive coupling can change the dynamic elastic constants, further modifying bandgap width and tunability. Therefore, the acoustic band structure is highly sensitive to the individual layer thicknesses, including Terfenol-D.

Therefore, when two Terfenol-D layers of different thickness sandwich a PMMA layer, the bandgap characteristics can be effectively tunned with respect to different magnetic field intensities, which is the main aim of this research.

The above-mentioned statements are briefly included in the 1^st^ paragraph under the section 2 of the revised manuscript.

**2. There is a vast literature on quasi-periodic phononic crystals. The presented literature review should position this work relative to the existing ones.**

We are thankful to the reviewer for this suggestion.

As per your suggestion, we have updated the introduction section by adding more recent works on quasi-periodic phononic crystal.

The characteristics of quasi-periodic phononic crystal and the recent research works are listed in the 2^nd^ and 3^rd^ paragraph under the introduction section of the revised manuscript.

**3. The historical background in the Introduction does not seem necessary for the context of the presented research.**

We are thankful to the reviewer for this suggestion.

We have omitted the historical background section from the introduction section in the revised manuscript.

**4. The authors present a realization with “three unit cells”. However, when discussing band diagrams, one refers to periodic structures. Why presenting a finite structure at this point?**

We appreciate the reviewer’s observation.

The band diagram is indeed defined for an infinite periodic structure; however, the inclusion of a finite structure with three unit cells serves a complementary purpose. The finite configuration is used to validate the practical realization of the design and to demonstrate transmission characteristics such as resonant modes within bandgaps. While the band structure provides theoretical insight into wave propagation, the finite model reflects experimentally feasible conditions and device-level behavior. Thus, presenting both allows bridging the gap between ideal periodic analysis and realistic implementation, ensuring that the predicted bandgap features effectively translate into observable sensing performance.

**5.** **“In the analysis of the transmission spectrum, Young’s modulus, density, and shear stress are critical parameters, as illustrated in figure 1”. Figure 1 does not deal with material parameters.**

We are really sorry for this mistake.

The Young’s modulus and density analysis of tungsten material and polycrystalline silicon material are illustrated in fig. 2. The detail description regarding this figure is stated in the 1^st^ paragraph under the section 3 of the revised manuscript.

Now, we have corrected this mistake in the revised manuscript.

**6. The authors explicitly mention the Poisson’s ratios for the two considered materials, but other material properties are not textually described.**

The authors appreciate the reviewer’s observation. We have mentioned all material properties like Young’s modulus, density and Poisson’s ration in the paragraph under the section 2.1 of the revised manuscript.

**7. The authors should include references for the interested reader concerning basic phenomena involving band gap formation (e.g., Bragg scattering) and the formulation of the mathematical model for obtaining transmission spectra.**

Thank you for the valuable suggestion. We agree that including references on the fundamental mechanisms of band gap formation will improve the clarity and completeness of the manuscript. Accordingly, we have revised the manuscript to incorporate key references that explain Bragg scattering and related wave interference effects responsible for phononic band gap formation in periodic and quasi-periodic structures. These references provide essential background for readers to better understand the physical origin of band gaps and their role in wave manipulation. The cited works have been added in the revised manuscript (Section X), ensuring that interested readers can readily access foundational concepts underlying the presented study.

With reference, the brief discussion on formation of band gap through Bragg scattering has been included in the 1^st^ paragraph under the section 202 of the revised manuscript.

The mathematical modelling for deriving the transmission spectrum is mentioned under the section 2.2 of the revised manuscript. Also, we have added the equation for obtaining the transmittance in equations 17 and 18 in the revised manuscript.

**8. Eq. (1) should be a part of a wider system of equations (including other directions). It is not clear what assumptions concerning the types of propagating waves and boundary conditions the authors have assumed.**

Ther authors are thankful to the reviewer for this suggestion.

Eq. (1) represents the scalar electrodynamic wave equation formulated under the assumption of one-dimensional longitudinal wave propagation normal to the layered interfaces. For completeness, it is implicitly part of a coupled system that includes transverse (shear) components; however. In our research, the shear modes are neglected due to the dominance of longitudinal waves in most propagated energy of acoustic waves in layered media. Since, most researches in phononic crystals prefer manipulation of acoustic wave and neglect shear waves as most energy of acoustic or elastic waves are dominated by the longitudinal modes [A.-Li. Chen, Y.-S. Wang / Physica B 392 (2007) 369–378]. Further, the stress-displacement relations are simplified using isotropic linear elasticity. The boundary conditions assume continuity of displacement and stress at each interface through the Transfer matrix method analysis that assumed in this researches and found as well in detail in previous literatures that already mentioned in the text as well. On the other hand, semi-infinite, non-reflecting media are considered at the outer boundaries. These assumptions are consistent with the transfer matrix framework.

We have included the assumptions concerning the types of propagating waves and boundary conditions in the paragraph below eq. 1 of the revised manuscript.

**9. Both lambda and mu refer to Lame parameters. However, mu is also recognized as the shear modulus (not shearing). Please verify along the manuscript if there are other typos.**

The authors are thankful to the reviewer for the clarification. We confirm that both λ and μ are Lamé parameters, where μ specifically represents the shear modulus. We have corrected this mistake in the revised manuscript.

**10. After Eq. (9), the authors mention a component of shear stress. But so far, the presented equations referred to the propagation of longitudinal waves. Please clarify.**

Thank you for this important observation. Firstly, the formulation and all TMM analysis considered general case of wave propagation but later in the discussion section we focused on longitudinal waves only for the previous mentioned reasons, so the state vector in Eqs. (8)–(9) is expressed in a generalized electrodynamic form, also, the shear stress term is corrected to the general case stress. Thanks for this important note. The general case stress should be more accurately interpreted as the relevant stress component associated with longitudinal motion (i.e., normal stress). Since mode conversion is neglected and only longitudinal waves are considered, shear wave contributions are not physically involved. The manuscript has been revised to clarify this distinction and avoid potential ambiguity.

**11. The authors mention “angle of incidence”, but the presented structure is a waveguide. Therefore, what does this angle refer to? If this involves incident acoustic waves, this is not clear from the discussion. In this case, also, the numerical methods utilized in the work should be discussed.**

The authors are thankful to the reviewer for this important observation. The term “angle of incidence” in this work refers to the angle between an externally excited plane acoustic wave and the normal to the layered interfaces, rather than a guided mode within a conventional waveguide. The structure is modeled as a stratified multilayer medium, where oblique incidence modifies the normal component of the wavevector, thereby influencing the Bragg condition and bandgap formation. This clarification has been inserted in the 1^st^ paragraph under the section 3 of the revised manuscript.

Regarding the numerical approach, the analysis is carried out using the transfer matrix method (TMM), which solves the 1D electrodynamic wave equation by enforcing continuity of displacement and stress at each interface. The global transfer matrix is constructed by cascading individual layer matrices, and the transmission coefficient is computed from its elements. The effect of incidence angle is incorporated through the wavevector decomposition, ensuring accurate modeling of oblique wave propagation.

**12. Where was the data from Fig. 2 obtained from? Did the authors use a reference or measure it?**

Thank you for the query.

The data presented in Fig. 2 were not experimentally measured in this work but were obtained from established literature sources reporting temperature-dependent material properties of tungsten and polycrystalline silicon. These datasets were then fitted using polynomial expressions for implementation in the simulations. Appropriate references are added in the revised manuscript to clearly acknowledge the data sources and ensure reproducibility.

**13. Figs. 3 and 4 seem to represent a parametric sweep (not an optimization, which involves setting an objective function, constraints, etc.).**

The authors are thankful to the reviewer for this suggestion.

The variations observed in Figs. 3 and 4 arise from systematic parametric changes in incident angle and layer thickness, which directly influence wave propagation and interference conditions within the phononic crystal. As the incident angle increases, the effective path length and phase accumulation of acoustic waves change, altering the Bragg condition and shifting the band gap positions and widths. Similarly, varying the thickness of the polycrystalline silicon layer modifies the acoustic impedance contrast and periodicity, leading to changes in constructive and destructive interference patterns. These effects result in the observed modulation of transmission characteristics and bandgap evolution, reflecting wave–structure interaction rather than formal optimization. Accordingly, we have modified the text in the revised manuscript.

**14. Figures 6-9 are only qualitatively described, but this should also be done quantitatively.**

The authors are thankful to the reviewer for this suggestion.

As per your suggestion, we have explained the figures 6-9 qualitatively as well as quantitatively in the respective places in the revised manuscript.

**Response to Reviewer 2's Comments:**

**1.   The abstract initially refers to “magnetic field sensing,” whereas the study focuses on temperature sensing. Can the authors clarify this inconsistency and clearly define the primary sensing mechanism investigated?**

The authors are very sorry for this mistake. This study mainly focuses on temperature sensing using band gap engineering in quasi-periodic phononic crystal. We have corrected any inconsistency in the revised manuscript.

**2. Several studies have already explored periodic and quasiperiodic 1D phononic crystals for sensing applications. What is the clear quantitative advancement of this work beyond existing literature, particularly regarding bandgap enhancement and sensitivity improvement?**

We are thankful to the reviewer for this suggestion.

Unlike prior studies that focus on a single quasiperiodic design or defect-based enhancement, this work provides a systematic comparative analysis across multiple quasiperiodic sequences under identical conditions. Additionally, the incorporation of temperature-dependent material properties and Monte Carlo-based robustness analysis further strengthens the practical relevance and distinguishes this work from existing literature. All of quasi and periodic structures are presented as a temperature sensor, so many comparisons and comprehensive analysis of all of them as a temperature sensor is presented. Further, the quantitative advancement of this work lies in both bandgap enhancement and sensitivity improvement within a unified material platform. Specifically, the Fibonacci quasiperiodic structure achieves a maximum bandgap width of **18.094×10⁶ Hz,** which represents a ~52% increase compared to the periodic structure **(11.889×10⁶ Hz).** In terms of sensing performance, a peak sensitivity of **−1175 Hz/K** is obtained for the Fibonacci configuration, which is significantly higher than the periodic case **(−584 Hz/K)** and exceeds several previously reported values for temperature-based PnC sensors.

The novelties of this research have been mentioned in the last paragraph of the introduction section of the revised manuscript.

**3.** **Why were tungsten and polycrystalline silicon specifically selected? Were alternative material combinations evaluated for higher acoustic impedance contrast or improved thermal stability?**

We are thankful to the reviewer for this suggestion.

Thank you for this important question. Tungsten and polycrystalline silicon were selected due to their significant acoustic impedance contrast, which is essential for strong Bragg scattering and wide phononic bandgap formation. Tungsten possesses high density and elastic modulus, while polycrystalline silicon exhibits comparatively lower density and stiffness, resulting in efficient wave confinement. Additionally, both materials demonstrate well-characterized and stable temperature-dependent properties, making them suitable for reliable thermal sensing analysis applications.

The above statements are included in the paragraph under the **section 2.1** of the revised manuscript.

From a practical perspective, polycrystalline silicon is widely used in MEMS fabrication, and tungsten offers excellent mechanical robustness and thermal stability, ensuring compatibility with existing fabrication technologies. While alternative material combinations (e.g., polymer-metal or ceramic-based systems) may provide higher impedance contrast, they often suffer from poor thermal stability or fabrication complexity. Therefore, this study prioritizes a balanced combination of performance, thermal reliability, and manufacturability, rather than solely maximizing impedance contrast.

**4. The temperature range extends from 293 K to 1673 K. Considering material limitations (e.g., oxidation, phase stability), how realistic is this range for practical implementation of the proposed sensor?**

We are thankful to the reviewer for this comment.

The temperature range of 293 K to 1673 K is considered primarily for theoretical and performance evaluation to understand the full thermal response of the proposed PnC structure. However, we agree that in practical implementations, the usable range would be more restricted due to material limitations such as oxidation of tungsten and structural changes in polycrystalline silicon at elevated temperatures.

Nevertheless, by using appropriate protective coatings like oxide or nitride layers, and maintaining a controlled environment of vacuum atmosphere, it is feasible to operate the proposed phononic crystal sensor at high temperatures.

**5.  The model assumes ideal interfaces and longitudinal wave propagation. How would interface roughness, mode conversion (shear waves), or thermoelastic damping affect the reported bandgap and sensitivity results?**

Thank you for this insightful comment.

The authors want to clarify that this research is based on theoretical modelling and analysis of the phononic crystal structure. To give a maximum focus on bandgap characteristic shift with respect to different operating temperatures, we have assumed an ideal interface condition.

However, in practical scenarios, interface roughness would introduce additional scattering and impedance mismatch variations, leading to slight bandgap broadening and reduced transmission contrast. Mode conversion may generate shear waves at interfaces, thereby weakening the longitudinal bandgaps. Further, the thermoelastic damping would introduce energy dissipation, reducing the quality factor and a slight degradation in the sensitivity. However, the fundamental bandgap formation and qualitative trends reported in this work are expected to remain valid, especially we maintain the angle after the optimization steps to avoid such conversion. Concerning, roughness, you completely right, this factor is a crucial factor especially in most nano scale designs like photonic crystals, but in our design we go to mm size to be far as much as we can from its effect. This high scale is beneficial as well for thermoelastic effect and make its effects roughly negligible.

Finally, we want to state that currently we are in a process of fabricating the proposed phononic crystal structure and we assure the reviewer that surface interface roughness and thermoelastic damping effect will be studied in the fabricated structure, and it will be reported in our upcoming work.

**6. Sensitivity is defined only based on bandgap width variation. Why was resonance frequency shift not considered, as it is commonly more precise for sensing applications?**

We are thankful to the reviewer for this insightful comment. In this work, sensitivity is defined based on bandgap width variation of periodic and quasiperiodic structures under different temperature variations. Moreover, the bandgap width provides a robust metric that is less sensitive to noise and fabrication imperfections.

However, we fully agree that resonance frequency shift is often more precise for sensing applications due to its sharp spectral features. Because in the quasiperiodic structure, different number of resonance peaks are achieved for different configurations like Fibonacci, Thue-Morse, double-periodic, and Cantor sequences. Additionally, tracking the shift in different resonance peaks is complicated, which will make the analysis complex.

We hope that we have satisfactorily answered this comment of the reviewer.

**7. The manuscript mentions 5% fabrication tolerances analyzed via Monte Carlo simulations. How many iterations were performed? Were statistical confidence intervals calculated? How do these uncertainties quantitatively impact sensitivity?**

Thank you for this important comment.

In this research, the Monte Carlo analysis is performed by using 1000 iterations to ensure statistical convergence of the transmission response. While explicit confidence intervals were not originally included, they can be readily derived from the obtained distributions (i.e. 95% confidence bounds).

Quantitatively, the introduced 5% uncertainties lead to minor bandgap shifts and slight broadening, resulting in a negligible sensitivity variation under 5% to 8%, which indicates that the proposed structure maintains robust sensing performance under realistic fabrication deviations.

**8. Although Fibonacci shows the widest bandgap, periodic structures show more stable sensitivity. Could the authors provide a normalized performance metric combining both bandwidth and stability to identify the optimal design objectively?**

Thanks a lot for this unique comment, surely in figures 15, 16 and discussion of table 1, the sensitivity of all designed structures versus temperature is given and discussed. We want to confirm on an important point, that the main object for each of these designs is the stability of sensitivity due to temperature changes, not the wideset band gap, as all designs are introduced as temperature sensor. So, in figures 15 and 16 we deduced that the perfect one was the optimum one and provide the highest sensitivity and deduced that is enough to clearly show our major task here. However, the discussion and metric below were added in the revised manuscript to clearly show the normalized performance metric combining both bandwidth and stability of all designs.

‘‘Lastly, we introduced a normalized performance metric (figure of merit, FOM) that combines both the bandgap width and the stability of sensitivity. The bandgap width was normalized with respect to the maximum observed value, while stability was quantified using the inverse (or normalized form) of the standard deviation of sensitivity over the considered temperature range.

Specifically, the combined metric is defined as:

${FOM}_{i}=\alpha\frac{B_{i}}{B_{max}}+(1-\alpha)(1-\frac{\sigma_{i}}{\sigma_{max}})$	(20)

where $B_{i}$ is the bandgap width, $\sigma_{i}$ is the standard deviation of sensitivity, $B_{i}$ is the bandgap width of structure *I*, $B_{max}$ is the maximum bandgap among all structures, and $\alpha$ is a weighting factor (taken as 0.5 for equal importance). Also, the normalized bandgap width $B^{*}$ is given as; $B^{*}=\frac{B_{i}}{B_{max}}.$ In addition, the stability of sensitivity is given as $S^{*}=1-\frac{\sigma_{i}}{\sigma_{max}}.$

This formulation enables an objective comparison between structures by simultaneously accounting for performance (bandgap width) and robustness (stability). Based on this metric, while the Fibonacci structure exhibits the largest bandgap, its relatively higher sensitivity fluctuations reduce its overall score. In contrast, the periodic structure shows excellent stability but a smaller bandgap.

Interestingly, the double-periodic structure emerges as an optimal compromise, achieving a balanced combination of reasonably wide bandgaps and improved stability compared to other quasiperiodic configurations. All normalized metrics of the designs are computed based on equation (20) and all inserted in table 2.

Table 2. Normalized Metrics of the performance of all suggested designs.

| Structure | Bandgap ($B^{*})$ | Stability ($S^{*})$ | ${FOM}_{i}$ |
| --- | --- | --- | --- |
| Periodic | 0.657 | 0.503 | 0.580 |
| Fibonacci | 1.000 | 0 | 0.500 |
| Thue-Morse | 0.701 | 0.366 | 0.534 |
| Double-period | 0.720 | 0404 | 0.562 |
| Cantor | 0.848 | 0.508 | 0.678 (BEST) |

**9. Figure 16 shows negative sensitivity values (e.g., −500 Hz/K to −1000 Hz/K). What is the physical interpretation of negative sensitivity in this context?**

We are thankful to the reviewer for this important question.

We want to clarify that the negative sensitivity values indicate that the measured parameter (i.e. bandgap width) decreases as temperature increases. Physically, this is attributed to thermal softening, where increasing temperature reduces the elastic modulus and slightly alters density, leading to a decrease in acoustic wave velocity and impedance contrast. Consequently, the band edges shift toward lower frequencies, resulting in a reduced bandgap width. Thus, negative sensitivity reflects an inverse temperature–response relationship, not a degradation in performance. Such behavior is typical in phononic systems and remains fully usable for sensing.

The above statements are included in the last paragraph under section 6 of the revised manuscript.

**10. The proposed structure involves millimeter-scale layers (e.g., up to 9 mm thickness). What fabrication technique is envisioned? Has scalability toward MEMS-compatible dimensions been considered?**

Thank you for this vital comment. The current design with millimeter-scale layers is intended for proof-of-concept and macroscale validation, where fabrication can be achieved using conventional techniques such as precision machining, layer stacking, diffusion bonding, or sputter deposition followed by wafer bonding. For practical sensing applications, especially MEMS integration, the structure can be scaled down proportionally since phononic bandgaps depend on the ratio of wavelength to lattice constant. Techniques such as thin-film deposition (CVD/PVD), lithography, and etching can be employed to realize microscale multilayers. So, with these advanced methodologies, it is feasible to fabricate the proposed design towards MEMS-compatible platforms. Another important point, the millimeter size of the lattice parameter, which is the most often used area in phononic crystal sensors and which also avoids the high GHz frequencies where structural relaxation.

The above statements are included under the section fabrication feasibility (section 8) of the revised manuscript.

**Response to Reviewer 3's Comments:**

This manuscript presents a comparative numerical investigation of temperature-dependent bandgap behavior in 1D tungsten/polycrystalline silicon phononic crystals, examining periodic and several quasiperiodic configurations (Fibonacci, Thue–Morse, double-periodic, and Cantor). The topic is relevant to acoustic wave engineering and sensing applications, and the attempt to systematically compare multiple deterministic aperiodic sequences under identical material platforms is commendable. The inclusion of fabrication tolerances and Monte Carlo–based robustness analysis adds practical value.

However, the manuscript in its current form requires substantial clarification and strengthening before it can be considered for publication.

**1. First, the novelty claim is somewhat overstated, as comparative studies of quasiperiodic phononic crystals for bandgap engineering are well represented in the literature; the authors should more clearly delineate what fundamentally new physical insight is provided beyond incremental parametric comparison.**

Thank you for this constructive comment.

We agree that quasiperiodic PnCs for bandgap engineering are well established in the literature. The novelty of this work is therefore not claimed in the mere use of quasi-periodicity, but in the new physical insight obtained through a unified, temperature-dependent comparative framework. Specifically, this study demonstrates a quantitative trade-off between bandgap enhancement and thermal stability, which has not been systematically addressed across multiple quasiperiodic sequences under identical conditions. In addition, in section 6, a new metric is presented in table 2 and new discussion as well is included to compare between all designs in their performance due to temperature changes.

A key contribution is the identification that Fibonacci ordering maximizes bandgap width (~52% enhancement) while periodic structures maintain superior stability and linearity of sensitivity, revealing a fundamental design trade-off for sensing applications. Additionally, the incorporation of temperature-dependent material dispersion and Monte Carlo-based robustness analysis provides deeper insight into real-world performance limitations.

The novelty statements are mentioned in the last paragraph of the introduction section in the revised manuscript.

**2. Second, the physical model lacks sufficient detail regarding boundary conditions, convergence testing, normalization procedures, and validation against either analytical limits or prior experimental/theoretical benchmarks.**

The authors are grateful to the reviewer for this comment.

In the simulation, the boundary conditions are defined by enforcing continuity of both displacement and normal stress at each material interface, by maintain consistency with the linear electrodynamic theory. The external boundaries are modeled as semi-infinite media, thereby eliminating artificial reflections, and ensuring physically realistic wave propagation.

Regarding numerical implementation, the convergence of the transfer matrix method has been carefully verified by systematically increasing the number of unit cells and confirming that the transmission spectra and bandgap characteristics exhibit negligible variation beyond a certain structural size.

The normalization procedure will be explicitly clarified, where frequency is expressed in a dimensionless form normalized with respect to lattice constant and acoustic wave velocity, enabling scalability and comparison across configurations.

The boundary conditions are mentioned in the 1^st^ paragraph under the section 2.2 of the revised manuscript.

**3. The temperature range up to 1673 K appears extremely high for polycrystalline silicon and tungsten multilayers, yet material stability and thermo-mechanical constraints are not discussed.**

We are thankful to the reviewer for this comment.

The temperature range of 293 K to 1673 K is considered primarily for theoretical and performance evaluation to understand the full thermal response of the proposed PnC structure. However, we agree that in practical implementations, the usable range would be more restricted due to material limitations such as oxidation of tungsten and structural changes in polycrystalline silicon at elevated temperatures.

However, we acknowledge that practical implementation may be constrained by thermo-mechanical stresses, interfacial diffusion, and oxidation, particularly for polycrystalline silicon and tungsten multilayers at elevated temperatures. In realistic applications, the operational range would be lower unless protective coatings of oxide or nitride layers, vacuum/inert environments, or advanced high-temperature fabrication techniques are employed.

This limitation has been included in the 2^nd^ paragraph under the section 8 of the revised manuscript.

**4. The definition of sensitivity based solely on bandgap width variation may oversimplify sensing performance; resonance frequency shift or Q-factor analysis would provide a more rigorous metric.**

We are thankful to the reviewer for this insightful comment. In this work, sensitivity is defined based on bandgap width variation of periodic and quasiperiodic structures under different temperature variations. Moreover, the bandgap width provides a robust metric that is less sensitive to noise and fabrication imperfections.

However, we fully agree that resonance frequency shift is often more precise for sensing applications due to its sharp spectral features. Because in the quasiperiodic structure, different number of resonance peaks are achieved for different configurations like Fibonacci, Thue-Morse, double-periodic, and Cantor sequences. Additionally, tracking the shift in different resonance peaks is complicated, which will make the analysis complex. In addition a new metric is presented in table 2 and new discussion as well is included in section 6 in order to compare between all designs through other performance parameters versus temperature changes.

We hope that we have satisfactorily answered this comment of the reviewer.

**5. Additionally, the manuscript contains significant language issues, repetition, and organizational redundancy that obscure the key findings.**

We are very sorry for these mistakes.

As per your suggestion, we have proofread the entire manuscript once again, and corrected the language issues, repetition, and organizational redundancy in the revised manuscript.

**6. Figures are extensively described but not critically interpreted in terms of physical mechanisms.**

We are thankful to the reviewer for this insightful comment.

We have added more explanations to different figures by focusing on the physical mechanism in the revised manuscript and as well new additions in the discussion section to enhance the performance section, besides the section of experimental feasibility. In the same time, we focused to add other important parts and calculations that asked by other reviewers and avoided not increasing the length of the manuscript and unnecessary verbosity that may reduce reader engagement.

**Finally, we hope this response meets the reviewers' comments and their points of view. In case the reviewers have any other comments, we are willing to carry out them all to match their requirements. Looking forward to hearing of acceptance of our contribution.**

**With respect in advance,**

**The corresponding author:**

**Hussein A. Elsayed**
